# Population pharmacokinetic and pharmacodynamic modeling of transformed binary effect data of triflusal in healthy Korean male volunteers: a randomized, open-label, multiple dose, crossover study

**DOI:** 10.1186/2050-6511-15-75

**Published:** 2014-12-23

**Authors:** Sung Min Park, Joomi Lee, Sook Jin Seong, Jong Gwang Park, Mi-Ri Gwon, Mi-sun Lim, Hae Won Lee, Young-Ran Yoon, Dong Heon Yang, Kwang-Il Kwon, Seunghoon Han

**Affiliations:** Clinical Trial Center, Kyungpook National University Hospital, Daegu, South Korea; Department of Biomedical Science, Kyungpook National University Graduate School, Daegu, South Korea; BK21 Plus KNU Bio-Medical Convergence Program for Creative Talent, Kyungpook National University Graduate School, 680 Gukchaebosang-ro, Jung-gu, Daegu, 700-842 South Korea; College of Pharmacy, Yeungnam University, Daegu, South Korea; Department of Internal Medicine, Division of Cardiology, Kyungpook National University School of Medicine, Daegu, South Korea; College of Pharmacy, Chungnam National University, Daejeon, South Korea; Department of Pharmacology, College of Medicine, The Catholic University of Korea, Seoul, South Korea; PIPET (Pharmacometrics Institute for Practical Education and Training), Seoul, South Korea

**Keywords:** HTB, Transformed binary data, Platelet aggregation, Binary probability model, NONMEM, Population pharmacokinetics and pharmacodynamics

## Abstract

**Background:**

Triflusal is a drug that inhibits platelet aggregation. In this study we investigated the dose-exposure-response relationship of a triflusal formulation by population pharmacokinetic (PK) and pharmacodynamic (PD) modeling of its main active metabolite, hydroxy-4-(trifluoromethyl) benzoic acid (HTB).

**Methods:**

This study was a randomized, open-label, multiple-dose, two-period, two-treatment, comparative crossover design. All volunteers received a single oral loading dose of 900 mg of triflusal on Day 1, followed by a dose of 600 mg/day from Day 2 to 9. Using data from 34 healthy volunteers, 476 HTB plasma concentration data points and 340 platelet aggregation data points were used to construct PK and PD models respectively using NONMEM (version 6.2). As the PD endpoint was qualitative, we implemented binary analysis of ‘inhibition’ and ‘non-inhibition’ rather than using the actual value of the test. The final PK-PD model was evaluated using a visual predictive check (VPC) and bootstrap.

**Results:**

The time-concentration profile of HTB over the entire dosing period was described by a one-compartment model with a first-order formation rate constant for HTB. Weight was selected as a covariate for clearance and volume of triflusal, respectively. The structure and the population estimates for triflusal PK were as follows: oral clearance (*CL/F*) = 0.2 · (weight/71.65)^0.845^ L/h, oral volume of distribution (*V/F*) = 8.3 · (weight/71.65) L, and *k*_*f*_ = 0.341 h^-1^. A sigmoid relationship between triflusal concentration and the probability of significant inhibition with shape factor was chosen as the final PD model. No time delay between concentration and response was identified. The final structure between predicted concentration  and the probability of inhibition of platelet aggregation (IPA) relationship was as follows: Probability of . Thus, we concluded this relationship is more like quantal concentration-response relationship. The current dosing regimen was considered to be efficacious based on the *EC*_*50*_ estimate of 84.9 μg/mL obtained in this study.

**Conclusions:**

A PK and binary probability PD model of triflusal was successfully developed for Korean healthy volunteers. The model may be used to further prediction inhibition of platelet aggregation by triflusal.

**Trial registration:**

Clinical Research Information Service (CRIS), KCT0001299 (Registered December 5, 2014)

## Background

Triflusal (2-acetoxy-4-(trifluoromethyl) benzoic acid), which is chemically related to salicylate [1] but is not a derivative of acetylsalicylic acid, is an antiplatelet drug [2, 3] that selectively inhibits arachidonic acid (AA) metabolism in platelets by irreversibly inhibiting cyclooxygenase-1 (COX-1) and reduces thromboxane B2 (TXB2) production [4, 5]. 2-Hydroxy-4-(trifluoromethyl) benzoic acid (HTB), the main active metabolite of triflusal, is formed by deacetylation upon passage through the liver [5]. Triflusal is less effective in inhibiting COX-1 and reducing TXB2 than aspirin, but more effective than HTB [4]. HTB increases the effects of triflusal on COX-1 inhibition compared with salicylic acid, the main metabolite of aspirin, which competes with the prodrug for the active site on the cyclooxygenase enzyme [5, 6].

Several previous reports have described the pharmacokinetic (PK) characteristics of triflusal. After oral administration, triflusal is absorbed rapidly within the small intestine, showing an absolute bioavailability of 83–100% [7]. The plasma half-life (*T*_1/2_) is 0.5 ± 0.1 h for triflusal and 34.4 ± 0.1 h for HTB [4]. More than 60% of the parent drug is eliminated by the kidney [7] and the values for renal clearance for triflusal and HTB were found to be 0.8 ± 0.2 L/h and 0.18 ± 0.04 L/h, respectively [5, 8]. HTB reaches steady-state levels after 8–10 days of treatment [5].

The aim of this study was to quantitatively determine the dose-exposure-response relationship of triflusal in healthy Korean male subjects using a population analysis. Additionally, we suggest a method of binary analysis that might be applied to platelet aggregation data to identify pharmacodynamic (PD) markers.

## Methods

### Inclusion and exclusion criteria

Volunteers aged 20–55 years and within 20% of their ideal body weight [IBW (kg) = height (cm) - 100) × 0.9] were enrolled in the study. Those who were not suitable on the basis of physical examinations and routine laboratory tests (blood hematology, biochemistry, prothrombin time, bleeding time, urinalysis) were excluded.

### Study design and ethical considerations

The analysis was performed using data from a randomized, open-label, multiple-dose, two-period, two-treatment, comparative crossover study involving 38 healthy adult males. The clinical trial was conducted at Kyungpook National University Hospital Clinical Trial Center (KNUH CTC) to determine the bioequivalence and non-inferiority of two different triflusal formulations, as reported previously [4].

The study was conducted in accordance with the guidelines of Good Clinical Practice and the Declaration of Helsinki and its amendments at the KNUH CTC, and was approved by the institutional review board at KNUH. Written informed consent was obtained from all volunteers before their participation.

### Study drugs and dosage regimen

Triflusal capsule (Disgren capsule, 300 mg), as the reference formulation, and triflusal EC capsule (Disgren enteric-coated capsule, 300 mg), as the test formulation were manufactured and provided by Myung-In Pharm. Co., Ltd (Seoul, Republic of Korea). The subjects received the test or reference formulation in multiple doses, followed by a 13-day washout period and subsequent administration of the alternative formulation. During each period, each subject received a single 900 mg oral loading dose on Day 1, followed by a 600 mg/day maintenance dose (given as two 300 mg capsules once daily) from Day 2 to 9.

### Blood sampling procedures

**Figure 1:**
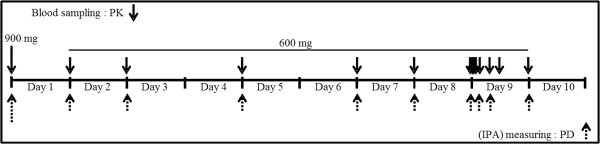
**PK and PD study designs.** Subjects (n = 34) eligible for this study were given a 900 mg loading dose on day 1, followed by a 600 mg/day maintenance dose on days 2, 3, 5, 7, 8, and 9. For PK assessment, blood samples were obtained at time 0, 24, 48, 96, 144, 168, 192, 192.5, 193, 194, 196, 199, 202 and 216 h. For PD assessment, blood samples were obtained at time 0, 24, 48, 96, 144, 168, 192, 196, 202, and 216 h. PK, pharmacokinetic; PD, pharmacodynamic; IPA, inhibition of platelet aggregation.

For measurement of the plasma concentration of HTB (PK), whole blood samples were obtained at time 0 (pre-dose), and 24, 48, 96, 144, 168, 192, 192.5, 193, 194, 196, 199, 202, and 216 h after the first dose (from Day 1 to Day 10). For platelet aggregation measurements (PD), sampling was performed at time 0 (pre-dose), and 24, 48, 96, 144, 168, 192, 196, 202, and 216 h post-dose. The overall sampling schema is presented in Figure 1.

Blood (7 mL) for PK analysis was collected into tubes containing sodium heparin, and blood (3 mL) for PD analysis was collected into tubes containing 0.109 mmol/L sodium citrate. An aliquot (1 mL) of each plasma sample was placed in a microcentrifuge tube containing 0.1 mL 0.1 M HCl to prevent degradation of triflusal in the plasma. The remaining samples were chilled promptly on crushed ice to maintain the distribution ratio between triflusal and HTB [9].

### HTB plasma concentration measurements

**Figure 2:**
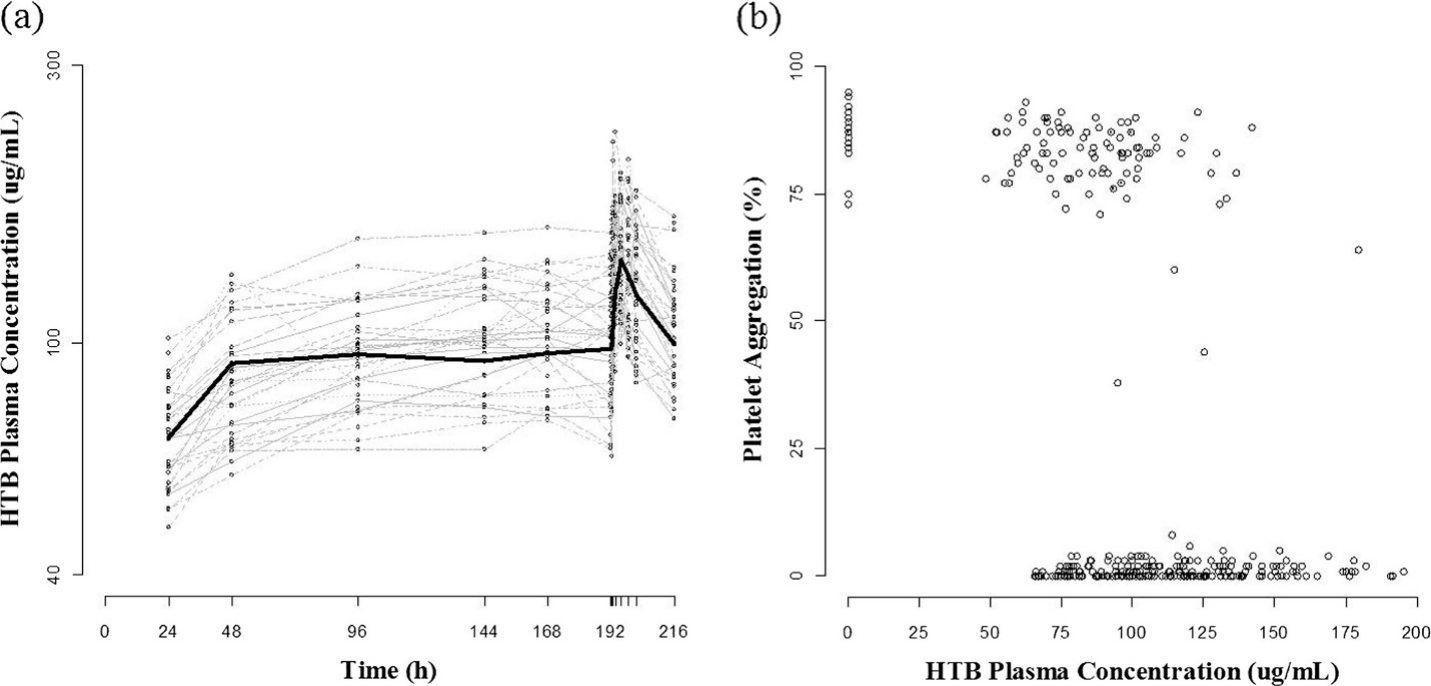
**Raw data plot of time-concentration-response profile. (a)** Profiles of observed individual and median HTB plasma concentration over time. **(b)** Observed concentration and platelet aggregation at the time of PD sampling.

Concentrations of HTB, rather than triflusal, were measured in this study because the level of triflusal is too low to be detected after 4 h when administered orally, as reported by Lee et al. [4, 10]. Blood samples were analyzed by high-performance liquid chromatography coupled with tandem mass spectrometry (HPLC-MS/MS) [7]. Quantification was performed by multiple reaction monitoring (MRM) transitions. A C18 column was used to separate the components of the samples by chromatography. Linear calibration curves were analyzed in the range of 1 to 300 μg/mL HTB (coefficient of correlation, r = 0.999). The lower limit of quantification of HTB was 1 μg/mL. The intra-day and inter-day % CVs for HTB were each less than 15%. Figure 2 shows the plasma concentrations of HTB with respect to time.

### Platelet aggregation assessment

The tubes containing sodium citrate were centrifuged (1,000 rpm, 10 min) and 500 μL of platelet-rich plasma (PRP) was collected as the supernatant. The PRP was transferred to microcentrifuge tubes, which were capped to prevent changes in pH upon exposure to air, and the samples were kept on ice. To obtain 500 μL of platelet-poor plasma (PPP), the remaining sample in the original tubes was centrifuged again (3,000 rpm, 10 min) [11]. Platelet aggregation in PRP was assessed using a Chronolog optical aggregometer (Model 490-4D; Chrono-log, Havertown, PA, USA). Cuvettes were lined up along the two rows of the aggregometer, filled with 250 μL of PRP or PPP, and warmed for 5 min at 37°C [12]. To measure the degree of platelet aggregation, 0.5 μL AA was added to the cuvettes as an agonist, and light transmission aggregometry was observed for 5 min. The aggregometer was calibrated with a cuvette containing PRP, equaling 0% light transmission, as the baseline and with a second cuvette containing PPP, equaling 100% light transmission, as the reference [13]. Based on analyses of the blank plasma samples, which were validated in three individuals, the intra-day and inter-day precisions were calculated as (SD/mean) × 100 (%) and ranged from 2.1 to 6.8% and from 2.1 to 7.6%, respectively [4].

### Dataset

In the current study, PK and PD data from 34 healthy volunteers who received the reference drug were used for population PK-PD analysis. In total, 476 HTB plasma concentration data points and 340 platelet aggregation (%) data points were included. The latent variables used as candidate covariates were age, height, and weight, and levels of albumin, creatinine, serum aspartate transaminase (*AST*), and alanine transaminase (*ALT*). Creatinine clearance (*CLCR*), calculated using the Cockcroft-Gault equation, was also included.

### Population PK-PD model development

The population PK-PD analysis was conducted by non-linear mixed effects modeling using NONMEM (ver. 6.2; Icon Development Solution, Ellicott City, MD, USA). The estimate and the between-subject variability for PK-PD parameters were investigated. The first-order conditional estimation (FOCE) method with interaction option was used whenever applicable [14]. The between-subject variability of each parameter of the basic model was applied using the exponential model:


where P_*ij*_ is the value of the *j*^th^ parameter in the *i*^th^ individual, θ_*j*_ is the typical value of the *j*^th^ population parameter, and η_*ij*_ is a random variable for the *i*^th^ individual in the *j*^th^ parameter. It is assumed that η_*ij*_ is normally distributed, with a mean of zero and a variance of ω^2^, and that η_*ij*_ ≈ N(0, ω_*ij*_^2^). The residual variability, consisting of intra-individual variability, experimental errors, process noise, and/or model mis-specifications, was modeled using additive (ϵ_*add,ij*_), proportional (ϵ_*pro,ij*_), and combined error structures. The combined additive and proportional error model on the following equation was initially applied to explain the gaps between the observed values and those predicted by individual PK parameters:


where C_*ij*_ is the *j*^th^ observed value in the *i*^th^ individual, and ϵ_*pro,ij*_ and ϵ_*add,ij*_ are the residual intra-individual variability with a mean of zero and variances of  and , respectively. A similar approach was made to the PD observations. The standard errors, which give information on the precision of the parameter estimates, were explored using NONMEM’s “$COVARIANCE” step.

Models were evaluated using diagnostic scatter plots, goodness-of-fit plots, and the log likelihood ratio test (LRT). The results for LRT were considered statistically significant if the objective function value (OFV) decreased by 3.84 (*p* = 0.05); this was used as a cut-off criterion for model improvement.

For PK, various absorption functions and distributional (1-compartment vs. multi-compartment) models were compared. When linking PK-PD relationship, two different models – binary probability and inhibition of build-up – were tried sequentially to find the best description on the PD data. The binary probability model uses a form of relationship similar to the Michaelis-Menten equation,  to explain instantaneous PK-PD relationship. In this equation, C_*pred,ij*_ is the estimated concentration over time for individuals, *EC*_*50*_ is the plasma concentration corresponding to the half-maximal response, and *γ* is the shape factor. To implement this approach, the PD data (% platelet aggregation) was transformed to a binary format based upon the finding that the distribution of PD observation (the maximum platelet aggregation observed in each sample) was clearly bimodal (Figure 2). The values above 74% were regarded as no inhibition of platelet aggregation (IPA), and thus as DV = 0, whereas values below 74 were regarded as IPA (DV = 1), according to a guideline presenting the normal ranges for the degree (%) of aggregation in platelet-rich plasma [15]. To find a reliable initial estimate for this model, a logistic regression procedure was performed in advance. For a binary output variable, Y, we can model conditional probability P(Y = 1 | X = x) as a function of x [16]. Let π(x) be a linear function of x, π(x) = P(Y = 1 | X = x). The model can be written as π(x) = exp(β_0_ + β_1_x) / exp(β_0_ + β_1_x) + 1. Equivalently, by a logit transformation, the logistic regression model has a linear relationship, logit(π(x)) = log(π(x) / 1 - π(x)) = β_0_ + β_1_x, where β_0_ is the intercept coefficient and β_1_ is the slope coefficient, for which the sign is positive or negative if π(x) is a strictly increasing or decreasing function of x, respectively [17]. Using the frequency of DV = 1 at each time point, the intercept (β_0_) and slope (β_1_) were estimated using SAS software (ver. 9.2; SAS Institute, Inc., Cary, NC, USA). The inhibition of build-up model was tried to evaluate whether there is a time-delay between concentration and response in the form of continuous variable. The differential equation for platelet aggregation at a certain HTB concentration is as follows [18]:


where dR/dt is the rate of change of the measured response (*R*) over time, *k*_*in*_ is the turnover input rate for production of the response, *I*_*max*_ is the fraction representing the maximal capacity by which the drug can inhibit platelet aggregation (0 ≤ *I*_*max*_ ≤ 1), *C* is the HTB concentration, *IC*_*50*_ is the median inhibition concentration, and *k*_*out*_ is the turnover output rate of the response.

### Covariate analysis

The parameter-covariate relationship, which explains why PK and PD vary among individuals, was explored using variables in the dataset. In the exploratory analysis, scatter plots of one covariate versus another were used to assess correlations between covariates and of parameters versus covariates. The numerical procedure used generalized additive modeling (GAM), as implemented in the Xpose library (ver. 4.4.0, Department of Pharmaceutical Biosciences of Uppsala University, Uppsala, Sweden) [19] of the ‘R’ software (ver. 2.15.3; R Foundation for Statistical Computing, Vienna, Austria). In this procedure, a response random variable, Y, is regressed on the individual covariates (X_j_) according to the general equation:


where the s_0_ is an intercept, and s_1_(X_1_), · · ·, s_p_(X_p_) are smooth functions that are estimated in a nonparametric fashion. In the equation, candidate covariates with the greatest decrease in the Akaike information criterion were selected as final covariates in the PK model when there was a reduction of at least 3.84 compared with the previous model, and the relationship between the random variable, η, for each parameter and the candidate covariates was improved. Otherwise, they were dropped from the model.

Graphical analyses using goodness-of-fit plots – including observed-versus-population-predicted, observed-versus-individual-predicted, and residual diagnostics – were also conducted to diagnose the degree of model development.

### Model evaluation

Bootstrap was recruited to check the robustness of the PK-PD model and parameter estimates. The median value and 95% confidence interval for each parameter were obtained from 1,000 bootstrap dataset. For the PK model, a visual predictive check (VPC) was implemented to further evaluate the accuracy and predictive performance. A total of 1,000 replicate simulation was performed and the 50th (estimated population median), 5th, and 95th percentiles of the created concentrations plotted against time were compared with the overlaid observed concentrations. In addition, predicted concentration-response relationship is plotted with the predicted probability of each concentration data observed at the time of PD sampling.

### Sample size determination

We considered the sample size for having an 80% power at a significance level of 0.05 for bioequivalence trials. It was calculated under the condition of the equivalence limit of 22.3%, the true mean difference of 10%, and the standard deviation of 24.2%. The calculation was performed by a formula suggested from Chow et al. [20]. Volunteers were randomly assigned to one of two sequences with simple randomization using SAS software (ver. 9.2; SAS Institute, Inc., Cary, NC, USA).

### Statistical analyses

The demographic data were presented using descriptive statistical analysis performed with the standard SPSS package (version 12.0 for Windows, SPSS, College Station, TX, USA). The mean, standard deviation, and range were obtained for each variable.

## Results

### Study population

**Figure 3:**
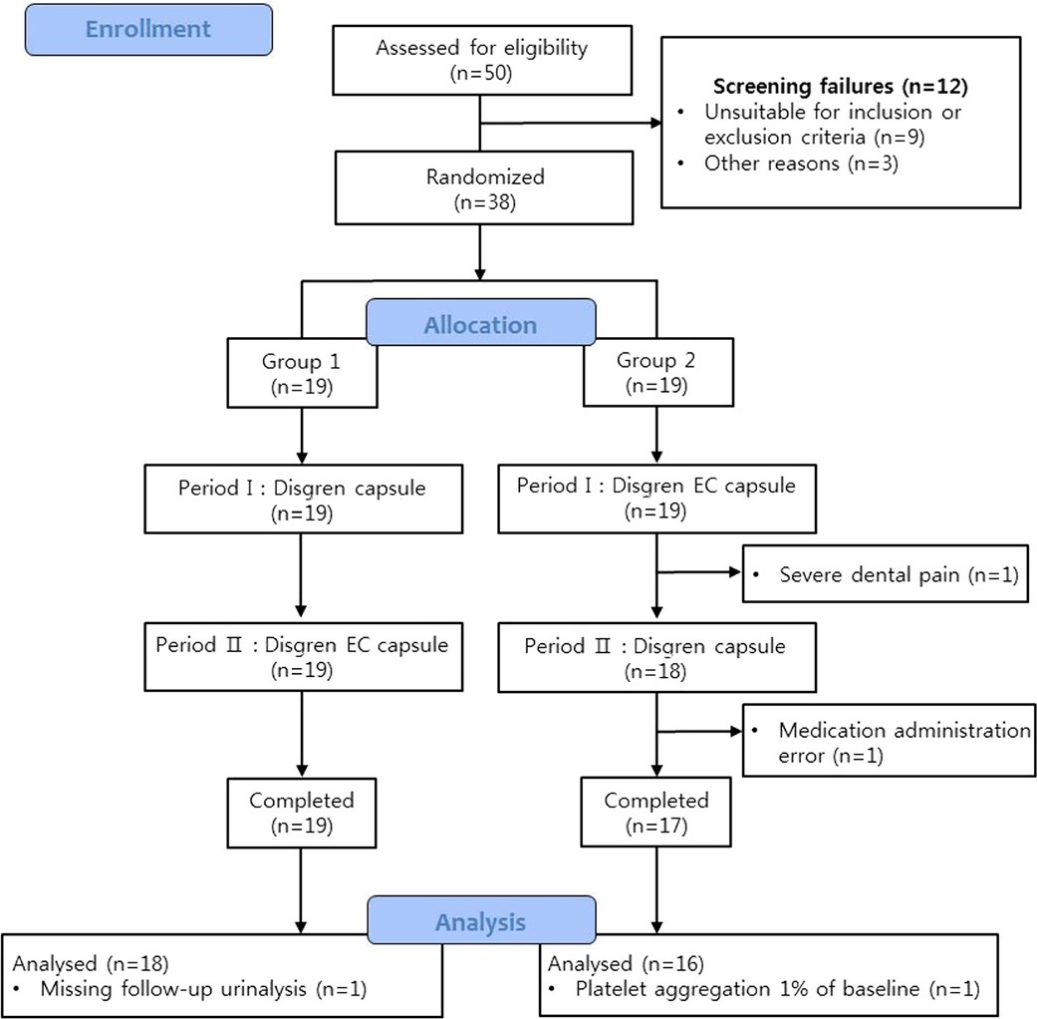
**CONSORT flow diagram of subject disposition.**

Volunteers were recruited from August 2008 to September 2008. In total, thirty-eight volunteers were enrolled in this study. Four subjects dropped out because of severe dental pain, a medication administration error, a missing follow-up urinalysis, or platelet aggregation 1% of baseline in the second period. Data from the remaining 34 subjects were analyzed to construct the PK and PD models. According to the CONSORT flow diagram, Figure 3 shows the subject disposition. The age, height, and weight (means ± SD) of the subjects were 24.1 ± 1.7 years, 176.1 ± 4.9 cm, and 70.8 ± 9.0 kg, respectively (Table 1).

**Table 1:** **Demographic information of the healthy volunteers**

Group	Number of subjects (n = 34)
	mean ± SD (Min – Max)
Age (years)	24.1 ± 1.7 (21 – 28)
Height (cm)	176.1 ± 4.9 (167.1 – 184.2)
Weight (kg)	70.8 ± 9.0 (53.3 – 89.7)

### Population PK modeling

The time-concentration profile of HTB over the entire multiple-dosing period was best described by a one-compartment model with first-order formation rate constant of HTB. This model was implemented in the PREDPP library subroutine “ADVAN2 TRANS2” in NONMEM. Residual variability was best described by the proportional error model, which explained the difference between the predicted and observed values for individuals and resulted in a significant decrease in the OFV. Weight was selected as the only covariate candidate after GAM analysis which explains existing knowledge about the PK of the drug. The effect of weight on *CL*/*F* and *V/F* was explained best with following each equation:


where θ_1_ and θ_2_ were the estimated typical values of *CL* and *V* with a median weight (71.65) and θ_4_ was the estimated influential factor for weight. Weight produced 30.782 (equivalent to *p* < 0.001, 1 degree of freedom) decrease in the minimized OFV as well as the change in the random BSV for *CL/F* from 18.5% to 14.9% and for *V/F* from 15.6% to 9.5% (as % CV). Because only one demographic variable (weight) was selected as a meaningful covariate, the process of backward covariate exclusion was omitted.

**Figure 4:**
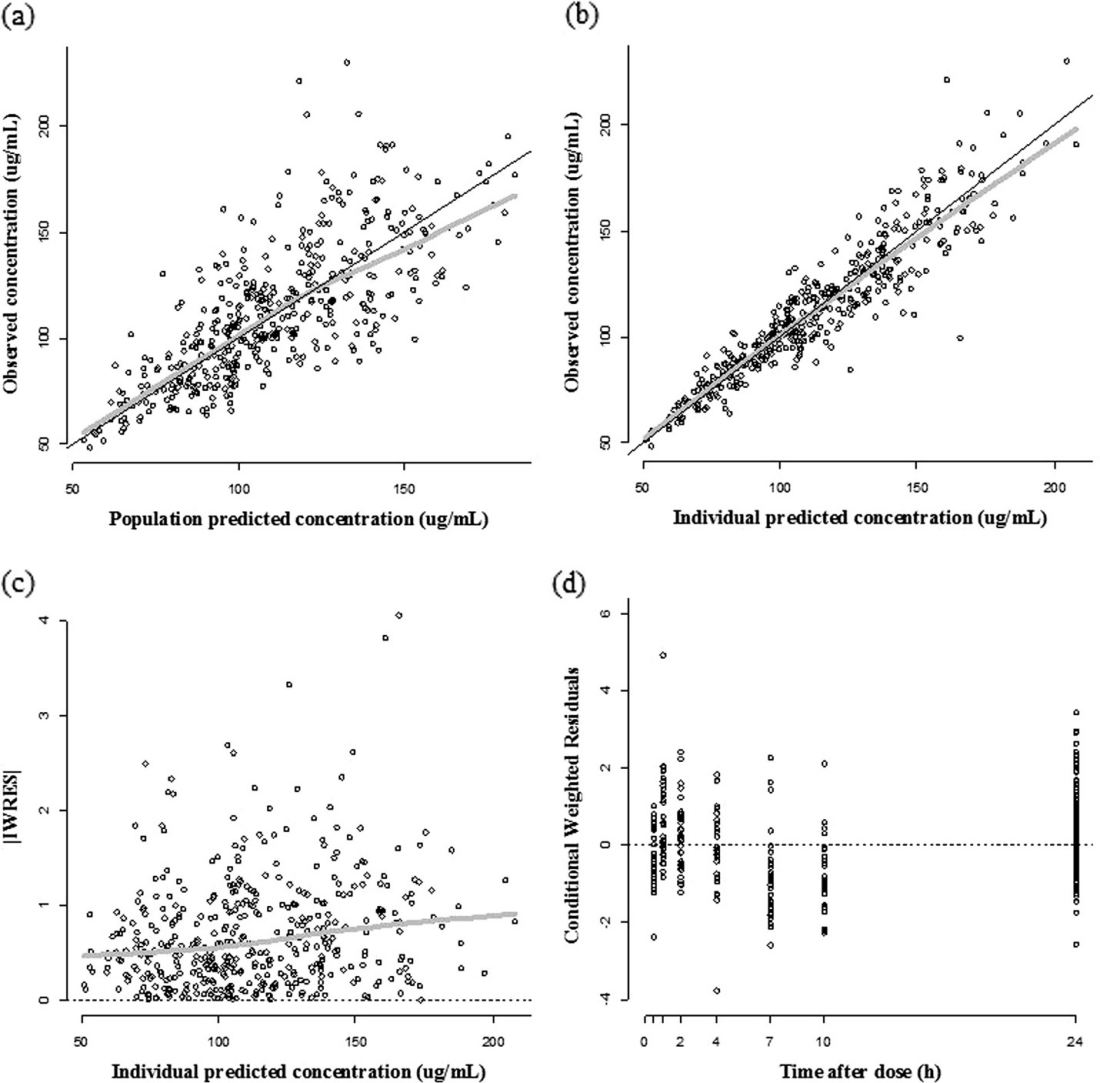
**Goodness-of-fit plots of the final population pharmacokinetic model. (a)** OBS versus PRED; **(b)** OBS versus IPRED; **(c)** |IWRES| versus IPRED; **(d)** CWRES versus time-after the last dose. Circle: observation; solid black line: line of identity; gray line: loess regression line. OBS, observation; PRED, population prediction; IPRED, individual prediction; |IWRES|, absolute individual weighted residuals; CWRED, conditional weighted residuals.

The goodness-of-fit plots for the final PK model are presented in Figure 4. Final estimates from the PK model, explaining the mean value and the between-subject variability, are listed in Table 2. The minimum concentration at steady state (*C*_*ss,min*_) was estimated to be 103.5 μg/mL using the values of final population PK parameters; in addition, the estimated value of the accumulation factor was 2.26.

**Table 2:** **Final estimates of population PK and PD parameters**

Parameter	Description (units)	Estimates from final model		Bootstrap results	Shrinkage (%)
		Estimate	% RSE ^a^	Median (95% CI)	
Fixed effect					
Pharmacokinetic					
*CL/F = θ*_*1*_ * (weight/71.65)^*θ4*^					
*θ*_*1*_	TV of *CL/F* (L/h) for subject whose weight = 71.65 kg	0.200	2.7	0.1998 (0.1995 – 0.2002)	
*θ*_*4*_	Exponent of weight proportional to *CL/F*	0.845	17.4	0.840 (0.830 – 0.850)	
*V*_*d*_/*F = θ*_*2*_ * (weight/71.65)					
*θ*_*2*_	TV of *V*_*d*_ (L) for subject whose weight = 71.65 kg	8.300	2.7	8.281 (8.267 – 8.295)	
*k*_*f*_ *= θ*_*3*_	TV of *k*_*f*_ (h^-1^)	0.341	15.1	0.345 (0.341 – 0.348)	
Pharmacodynamic					
*θ*_*5*_	TV of *EC*_*50*_ (μg/mL)	84.9	4.0	85.19 (84.98 – 85.40)	
*θ*_*6*_	TV of *γ*	19.2	22.4	20.70 (20.32 – 21.08)	
BSV (%CV)					
Pharmacokinetic					
*ω*_*1*_^*2*^	BSV for *CL/F*	14.9	21.0	14.4 (14.3 – 14.5)	0.6
*ω*_*2*_^*2*^	BSV for *V*_*d*_	9.5	57.8	8.6 (8.4 – 8.8)	40.2
*ω*_*3*_^*2*^	BSV for *k*_*f*_	88.0	26.5	73.5 (72.8 – 74.1)	10.5
Pharmacodynamic					
*ω*_*4*_^*2*^	BSV for *EC*_*50*_	21.8	28.4	21.4 (21.2 – 21.5)	8.3
*ω*_*5*_^*2*^	BSV for *γ*	0 (Fixed)	NE	-	-
Residual error (σ^2^)^b^					
Proportional error		0.098	6.5	0.0977 (0.0973 – 0.0981)	11.5

% RSE, % relative standard error; CI, confidence interval; *CL/F*, oral clearance; TV, typical value at population level; *V*_*d*_ */F*, oral volume of distribution; *k*_*f*_, first-order formation rate constant; *EC*_*50*_, HTB concentration at which the probability of IPA is 50%; *γ*, shape factor; BSV, between-subject variability; % CV, % coefficient of variation; NE, not estimated.

^a^% RSE is calculated from ω^2^.

^b^Additive residual error was not selected.

### Population PD modeling

The binary probability model was selected as the final PK-PD structure. The results of the estimation from this PD model were as follows: *γ* = 19 and *EC*_*50*_ = 84.9 μg/mL. In addition to these parameter estimates, information such as the observed and predicted number of times that non-aggregation occurred at each time point is shown in Table 3. The between-subject variability was only applied to *EC*_*50*_ and was well explained. The values estimated from the PD model are summarized in Table 2.

**Table 3:** **Observed and predicted IPA over time**

Time	Number of subjects	Number of observed IPA (%)	Number of predicted IPA (%)
0 h	34	1 (3%)	0 (0%)
24 h	34	2 (6%)	2 (6%)
48 h	34	11 (32%)	11 (32%)
96 h	34	29 (85%)	25 (74%)
144 h	34	25 (74%)	28 (82%)
168 h	34	27 (79%)	30 (88%)
192 h	34	30 (88%)	30 (88%)
196 h	34	34 (100%)	34 (100%)
202 h	34	34 (100%)	34 (100%)
216 h	34	28 (82%)	30 (88%)

IPA, inhibition of platelet aggregation.

### Model evaluation

**Figure 5:**
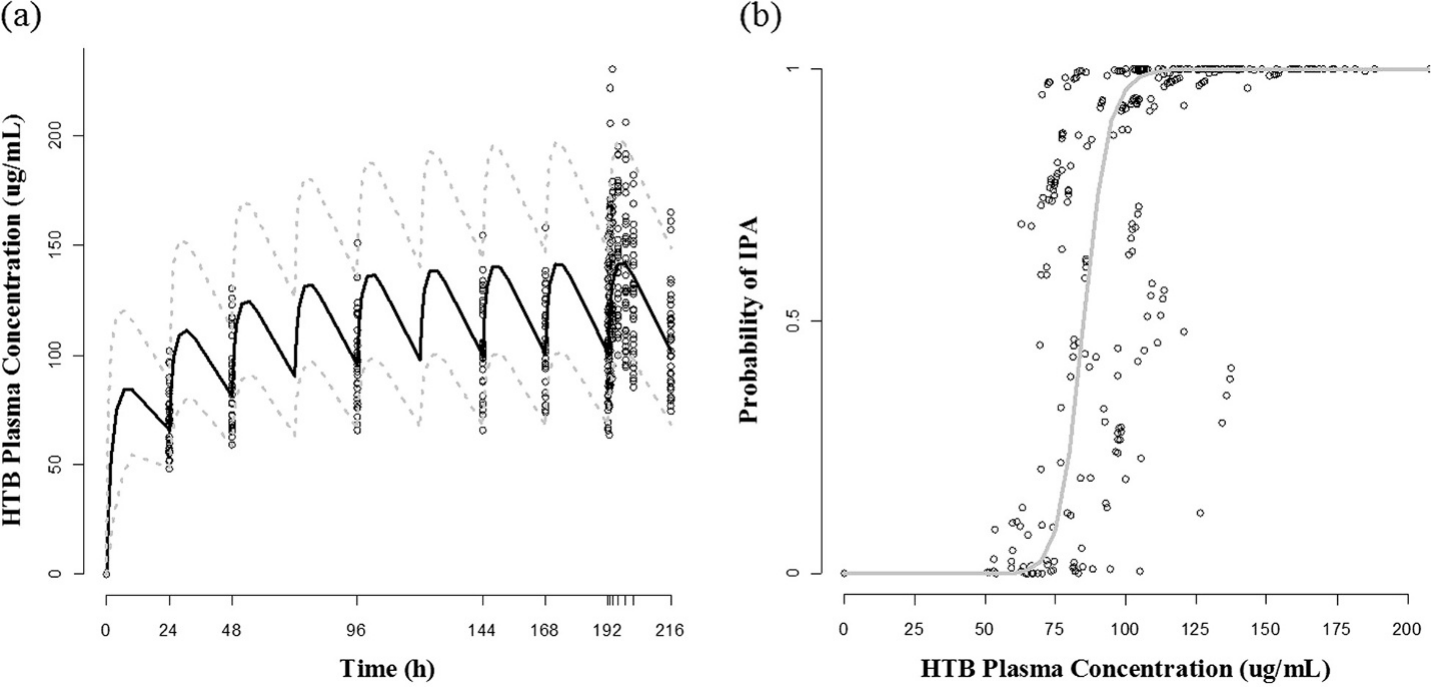
**Results of model evaluation. (a)** Visual predictive check result. The 5th and 95th percentiles (dashed gray line) and the median value (solid black line) from the simulated data are plotted against the observed concentration data (circle) according to time. **(b)** Predicted HTB concentration-probability of IPA curve; Circle: expected IPA for each HTB concentration measured at the time of PD observation; IPA, inhibition of platelet aggregation.

The bootstrap results showed acceptable robustness of the model and are presented together with the final estimates in Table 2. The VPC result (Figure 5) showed that the prediction of the simulated data well-matched the observed concentration-time profiles. This graph, representing a visual internal validation of the model, showed that most of the observed data points were overlaid between the 5th and 95th percentiles.

## Discussion

The PK and PD characteristics of triflusal were investigated using a non-linear mixed effects analysis. The objective of this study was to develop a population PK and PD model of the triflusal formulation, an established uncoated capsule, in healthy Korean male subjects. Plasma concentrations of the active metabolite, HTB, and the degree of platelet aggregation inhibition were measured to demonstrate the PK and PD properties. Reasonable estimates for parameters of the PK model were obtained, reflecting the potential for accurately predicting the information sought. The plots of the observed concentrations versus population-predicted or individual-predicted concentrations showed that the PK model was well structured, and the plot of time versus conditional weighted residuals suggested that the errors had homogeneous variances.

Our final HTB PK structure includes first-order formation and 1-compartment disposition. This is plausible judging from the immediate increase in HTB concentration on the day of full-PK study after dosing time and the rate of concentration increase which is the fastest at immediate post-dose and decreases thereafter (Figure 2). This finding is consistent to the model used by Valle et al. [3]. On the contrary, Yun et al. [21] suggested a 2-compartment model for HTB disposition. But we considered this model would behavior like a 1-compartment model because the distributional rate constant between compartments were much (approximately 60 times) larger than the elimination rate constant and the equilibrium between compartments will be obtained within 1 h after dose. Judging from the time of maximum plasma concentration attainment (postdose 4–5 h), this distributional characteristic might be superimposed by the absorption as well as the elimination.

Covariate model building was established by the relationship between the subjects’ body features and parameters in the PK model. Weight accounted for approximately 19 % and 39 % of between-subject variability for *CL/F* and *V/F*, respectively, judging from the decrease of ω^2^ for corresponding parameters. Particularly, *V/F* was directly proportional to weight (exponent = 1) and the fact suggested that weight may be used as a scaling factor for triflusal dose. However, definite conclusions could not be made for following reason; 1) this study did not consider the PK of triflusal; thus, weight might be influential to the disposition of triflusal including metabolic fraction to HTB (which was only explained with *F* in this study), 2) the clinical significance of this finding needs further investigations together with its pharmacodynamics variability, therapeutic index and clinical effectiveness. In addition, even though HTB is mainly (>60%) excreted in urine, *CLCR* was not selected as a meaningful covariate for HTB *CL/F*. We consider that the effect of weight superimposed that of *CLCR*. It was expected that studies involving triflusal and HTB PK in patients with various renal function will be essential to elucidate the exact influence of renal function.

Based on the value of *EC*_*50*_ (=84.9 μg/mL) estimated in this study, the current dosing regimen was considered to be efficacious judging from the *C*_*ss,min*_ value (=103.5 μg/mL) and the concentration-response relationship which showed quantal characteristics (γ = 19). The saturated value of *E*_*max*_ as 1 seemed reasonable, because there was no significant model improvement when the parameter was allowed to be estimated.

Another method, the binary probability model to construct the PD model using transformed binary data, was introduced to show characteristics of the PD data without much information loss. This model resulted in successful explanation of binary data characteristics by the estimates, even though a turnover model was not constructed because of the modified binary data characteristics. To date, this is the first report evaluating quantitative PD data transformed into qualitative binary data possessing one of two values. We recommend this new approach of using probability to estimate parameters that show predictive ability for the presence of platelet aggregation. This model also provided a specific method for potential use with binary data in the case of high intra-individual variability for PD.

This study has some limitations. First, only HTB concentrations were used in our population PK and PD model without measurement of the parent drug. Although triflusal is a prodrug which has no pharmacologic effect, if a study involving the concentration measurement of both triflusal and HTB is conducted, a better description for the HTB formation will be obtained. This approach may enable the identification of influential factors for HTB formation process including first-pass metabolism and hepatic clearance of triflusal. Second, despite the possible loss of information from the data transformation, a PD model using probability (binary data model) was developed to evaluate the extreme values in our PD data, as seen in the scatter diagram. To overcome this problem, more accurate and precise quantitative analytic method to measure the degree of platelet aggregation should be required.

Using the VPC, which is one method of model evaluation, prediction is possible when given not only different dosage- and time-concentrations but also the probability of IPA. Accordingly, optimal dosage regimens could be determined by model-fitted parameter estimates without additional clinical trials. The model may be clinically useful for other similar studies. Finally, this study was conducted in healthy volunteers, rather than in patients. Thus, it is also necessary to construct a model estimating reasonable parameters from patients in a more realistic setting.

## Conclusions

A PK and binary probability PD model of triflusal was successfully developed for Korean healthy volunteers. The model may be used to further predict inhibition of platelet aggregation by triflusal.

## References

[1] Matias-Guiu J, Ferro JM, Alvarez-Sabin J, Torres F, Jiménez MD, Lago A, Melo T (2003). Comparison of triflusal and aspirin for prevention of vascular events in patients after cerebral infarction: the TACIP Study: a randomized, double-blind, multicenter trial. Stroke.

[2] McNeely W, Goa KL (1998). Triflusal. Drugs.

[3] Valle M, Barbanoj MJ, Donner A, Izquierdo I, Herranz U, Klein N, Eichler HG, Müller M, Brunner M (2005). Access of HTB, main metabolite of triflusal, to cerebrospinal fluid in healthy volunteers. Eur J Clin Pharmacol.

[4] Lee HW, Lim MS, Seong SJ, Lee J, Park J, Seo JJ, Yun HY, Baek IH, Kwon KI, Yoon YR (2011). A phase I study to characterize the multiple-dose pharmacokinetics, pharmacodynamics and safety of new enteric-coated triflusal formulations in healthy male volunteers. Expert Opin Drug Metab Toxicol.

[5] Anninos H, Andrikopoulos G, Pastromas S, Sakellariou D, Theodorakis G, Vardas P (2009). Triflusal: an old drug in modern antiplatelet therapy. Review of its action, use, safety and effectiveness. Hellenic J Cardiol.

[6] Rao GH, Reddy KR, White JG (1982). Effect of acetaminophen and salicylate on aspirin-induced inhibition of human platelet cyclo-oxygenase. Prostaglandins Leukot Med.

[7] Murdoch D, Plosker GL (2006). Triflusal: a review of its use in cerebral infarction and myocardial infarction, and as thromboprophylaxis in atrial fibrillation. Drugs.

[8] Gonzalez-Correa JA, De La Cruz JP (2006). Triflusal: an antiplatelet drug with a neuroprotective effect?. Cardiovasc Drug Rev.

[9] Cho HY, Jeong TJ, Lee YB (2003). Simultaneous determination of triflusal and its major active metabolite, 2-hydroxy-4-trifluoromethyl benzoic acid, in rat and human plasma by high-performance liquid chromatography. J Chromatogr B Analyt Technol Biomed Life Sci.

[10] Ramis J, Mis R, Forn J, Torrent J, Gorina E, Jane F (1991). Pharmacokinetics of triflusal and its main metabolite HTB in healthy subjects following a single oral dose. Eur J Drug Metab Pharmacokinet.

[11] Dong HP, Wu HM, Chen SJ, Chen CY (2013). The effect of butanolides from Cinnamomum tenuifolium on platelet aggregation. Molecules.

[12] Harrison P, Mackie I, Mumford A, Briggs C, Liesner R, Winter M, Machin S (2011). Guidelines for the laboratory investigation of heritable disorders of platelet function. Br J Haematol.

[13] Zhou L, Schmaier AH (2005). Platelet aggregation testing in platelet-rich plasma: description of procedures with the aim to develop standards in the field. Am J Clin Pathol.

[14] Beal S, Sheiner L (1992). NONMEM User’s Guide Part I.

[15] Chrono-log corporation (2005). Instruction manual for the chrono-log model 490 optical aggregometers.

[16] Agresti A (2002). Logistic Regression. Categorical Data Analysis.

[17] Casella G, Berger RL (2001). Regression Models. Statistical Inference.

[18] Gabrielsson J, Weiner D (2007). Pharmacodynamic Concepts. Pharmacokinetic and Pharmacodynamic Data Analysis: Concepts and Applications.

[19] Jonsson EN, Karlsson MO (1999). Xpose–an S-PLUS based population pharmacokinetic/pharmacodynamic model building aid for NONMEM. Comput Methods Programs Biomed.

[20] Chow SC, Wang H (2001). On sample size calculation in bioequivalence trials. J Pharmacokinet Pharmacodyn.

[21] Yun HY, Kang W, Lee BY, Park S, Yoon YR, Yeul Ma J, Kwon KI (2014). Semi-Mechanistic Modelling and Simulation of Inhibition of Platelet Aggregation by Antiplatelet Agents. Basic Clin Pharmacol Toxicol.

[CR22] The pre-publication history for this paper can be accessed here: http://www.biomedcentral.com/2050-6511/15/75/prepub

